# Predictors of Time to Relapse/Recurrence after Electroconvulsive Therapy in Patients with Major Depressive Disorder: A Population-Based Cohort Study

**DOI:** 10.1155/2011/470985

**Published:** 2011-11-03

**Authors:** Axel Nordenskjöld, Lars von Knorring, Ingemar Engström

**Affiliations:** ^1^Department of Psychiatry, Örebro County Council, Örebro University Hospital, 701 85 Örebro, Sweden; ^2^Psychiatric Research Centre, School of Health and Medical Sciences, Örebro University, 701 82 Örebro, Sweden; ^3^Department of Neuroscience, Psychiatry, Uppsala University, 751 85 Uppsala, Sweden

## Abstract

*Objective*. The aim of the study is to define predictors of relapse/recurrence after electroconvulsive therapy, ECT, for patients with major depressive disorder. *Methods*. A study of all patients (*n* = 486) treated by means of ECT for major depressive disorder was performed. The data were derived from a regional quality register in Sweden. Psychiatric hospitalisation or suicide was used as a marker for relapse/recurrence. *Results*. The relapse/recurrence rate within one year after ECT was 34%. Factors associated with increased risk of relapse/recurrence included comorbid substance dependence and treatment with benzodiazepines or antipsychotics during the follow-up period. *Conclusions*. Within the first years after ECT, relapses/recurrences leading to hospitalisation or suicide are common. Treatment with lithium might be beneficial, while benzodiazepines, antipsychotics, or continuation ECT does not seem to significantly reduce the risk of relapse/recurrence.

## 1. Introduction

Depression is a major public health concern [1]. In severe depression, more than 70% of the patients experience repeated relapses/recurrences, chronicity or commit suicide [2, 3]. Among patients treated as inpatients, the risk for rehospitalisation in the first year exceeds 30% [4, 5]. 

Electroconvulsive therapy, ECT, is an effective acute treatment in severe forms of depression, such as psychotic or catatonic depression. ECT has also been recommended in less severe forms of depression after pharmacotherapeutic failure [6]. However, the rate of relapses/recurrences is high. 

Thus, a series of treatment strategies during the follow-up period has been tested to reduce the risk of relapse/recurrence. One alternative is antidepressants, commonly used for relapse/recurrence prevention after ECT. Also different augmentation strategies have been suggested. In a randomised trial, a lithium-antidepressant combination was found to be more effective than antidepressants alone in preventing relapses/recurrences after ECT [7]. Continuation ECT is becoming increasingly used to reduce the relapse/recurrence rate and is supported by a randomised trial in which continuation ECT alone and an antidepressant-lithium combination resulted in similar relapse/recurrence rates [8]. Despite these treatments, relapse/recurrence rates of 40–50% within 6–12 months after ECT have been reported [7–9]. 

Some second-generation antipsychotics have also been demonstrated to be effective in the acute treatment of major depressive disorder [10, 11], and a few studies suggest effectiveness in relapse prevention of major depressive disorder [12]. However, more data on the use of antipsychotics for the prevention of relapses/recurrences after ECT are needed.

In major depression, a greater number of depressive episodes tend to increase the risk of subsequent relapses/recurrences [13]. Patients with diseases resistant to medication have an increased risk of relapse/recurrence after ECT [14]. In parallel, in several studies of patients treated for depression with medication, remaining symptoms were correlated to increased risk of relapse/recurrence [13, 15]. Patients with comorbid conditions may also run an increased risk of relapse/recurrence [16, 17]. In some studies severe symptoms were associated with increased risk of relapse/recurrence [18].

The aim of the present study is to define predictors of time to relapse after ECT for major depressive disorder, single or recurrent, in a consecutive sample of patients from a defined geographical area in Sweden.

## 2. Patients and Methods

### 2.1. The Regional Quality Register for ECT

In Sweden, all ECTs are provided in psychiatric hospitals responsible for the treatment of all patients in a defined geographical area. All inhabitants are assigned a personal identification number that is used in the hospital charts and in the computerized administrative systems. Therefore, all inhabitants in a designated area treated with ECT can be identified. Seven hospitals in the middle of Sweden collaborate and report clinical data to a regional quality register for ECT. The data in this study were derived from the register. Data about personal registration number, diagnoses, number of ECT sessions, treatment parameters, clinical global impression-improvement (CGI-I) [19], Montgomery Åsberg Depression Rating Scale-Self-assessment (MADRS-S) [20], pharmacotherapy, and continuation ECT were reported at the conclusion of each index series of ECT. The psychiatrist in charge of each patient determined the diagnosis. Experienced nurses who met the patients during the ECT assessed CGI-I. The outcomes were graded as very much improved, much improved, minimally improved, or not improved. A nurse monitored the register for completeness and visited each hospital every three months to check the data registered with the data in the hospital charts and patient-administrative systems.

### 2.2. Subjects

Inclusion criteria for the study were

1. treatment with ECT in one of the seven hospitals in the middle of Sweden between January 1, 2008 and December 31, 2009,
2. a diagnosis of major depressive disorder, single episode or recurrent.

Exclusion criteria were

1. bipolar disorder,
2. schizoaffective disorder,
3. schizophrenia.

### 2.3. ECT Treatment

ECT was administered using a bidirectional constant current, brief pulse device. The Mecta Spectrum 5000Q device (Mecta Corp, Lake Oswego, Ore) was used at five hospitals, and a Thymatron system IV (Somatics, Inc, Lake Bluff, Ill) was used at two hospitals. 

The mean number of ECT sessions was 7.9 ± 3.0. Most treatments were unilateral, but in 12% at least one of the treatments in the series was bitemporal. The mean dosage at the last treatment if unilateral was 0.46 ± 0.10 ms, 75 ± 25 Hz, 7.5 ± 0.9 s, 833 ± 50 mA, and 429 ± 177 mC. More than 20 seconds of EEG-monitored effect was sought. The mean EEG seizure duration was 34 ± 15 s. 

Propofol (mean dosage 104 ± 40 mg) or thiopental (mean dosage 288 ± 98 mg) was used as anaesthetic. Succinylcholine (1 mg/kg) was used as muscle relaxant, and glycopyrrolate (0.2 mg) was used as anticholinergic. If no adverse events occurred, ECT was continued until the patients were asymptomatic or the physician judged that the patient had benefitted as much as possible. 

For patients receiving continuation ECT, the mean number of continuation ECT sessions was 12 ± 15.

### 2.4. Follow-Up Data

Follow-up data were reported to the register during the summer/autumn 2010. All follow-up data from a specific hospital were collected on the same date.

### 2.5. Outcome

In the present study, time to psychiatric hospitalisation or death due to suicide was used as a robust indicator of a relapse/recurrence. Thus, hospitalisation or suicides were the main outcome measures. The starting point was the last day of the first index ECT series in the period and the endpoint the first day of hospitalisation in psychiatric care or time of suicide.

### 2.6. Statistics

The cumulative probability of surviving without psychiatric hospitalisation was calculated with Kaplan-Meier technique. Differences in time to relapse were tested using univariate Cox regression. In order to assess the relative importance of different factors, a Cox regression analysis was performed. Time to relapse was the dependent variable, and independent variables with a trend toward statistical significance (*P* < 0.10) in the univariate analyses were entered. The tests performed were two sided, and alpha was set to 0.05. SPSS version 15.0 (SPSS Inc, Chicago, Ill) was used for the statistical analyses.

### 2.7. Ethics

The Regional Ethical Vetting Board in Uppsala approved the study. The patients were informed about the register and accepted registration.

## 3. Results

### 3.1. Participants

During the period, 487 patients were treated with ECT for depression. 

The subjects are described in Table 1. 56.5% were women. The mean age was 55 ± 18 years. 77.7% had major depressive disorder, recurrent episodes. 86.8% were in voluntary treatment, and 75.4% were treated as inpatients. 29.5% had a comorbid anxiety disorder, and 10.4% had comorbid substance dependence. 7.5% had a personality disorder. 

Information about hospitalisations and continuation ECT was available for patients living in the area. Only one patient was excluded due to lack of follow-up data. 15 patients migrating from the area were censored in the statistical analysis at the day of migration. Six patients that died from somatic diseases were censored at the day of death. Information about alive/dead status was available for all patients. The national causes of death registry provided causes of death data for all deaths. 

The median follow-up time was 564 days (range 173–1128 days).

### 3.2. Psychiatric Hospitalisation during Follow-Up

Out of 486 patients, 185 (38.1%) were hospitalised for psychiatric reasons. The cumulative proportions of the total sample of patients with relapses/recurrences during six months, one year, and two years were 25%, 34%, and 44%, respectively. 

The cumulative proportions of inpatients with relapses/recurrences were 29%, 39%, and 51% during six months, one year, and two years, respectively. The cumulative proportions of outpatients with relapses/recurrences were 12%, 19%, and 25% during six months, one year, and two years, respectively.

The main diagnoses at hospitalisation during followup were depression 90%, borderline personality disorder 3%, substance dependence 3%, and anxiety disorders 4%. None of the patients were hospitalised due to mania.

### 3.3. Mortality

Fifteen patients died during the follow-up period. Nine patients died from suicide and 6 from somatic diseases. The cumulative proportion of patients dying from suicide within one year after ECT was 2%. None of the patients died from complications of ECT.

### 3.4. Age, Sex, and Diagnosis

Neither sex nor age significantly influenced the rate of relapse/recurrence (Table 2). 

Patients treated for major depressive disorder, recurrent episodes had a similar relapse rate as patients treated for the major depressive disorder, single episode (Table 2). 

The severity of the depressive episode was not significantly related to the relapse/recurrence rate (Table 2). 

A higher proportion of patients with comorbid alcohol or illicit substance dependence had relapse/recurrence as compared to patients without such diagnoses (Table 2). The main diagnosis was major depressive disorder, recurrent episodes in 21 (77.8%) out of 27 hospitalisations during followup among patients with substance dependence.

### 3.5. Symptoms after Index ECT

Symptom control after ECT as assessed by means of CGI-I did not significantly influence the rate of relapse/recurrence (Table 2).

### 3.6. Treatment Factors

#### 3.6.1. Lithium during the Follow-Up Period

Patients receiving lithium after ECT seemed to have a reduced risk of relapse as compared to patients without lithium treatment (HR = 0.51, *P* = 0.03) (Table 3). However, the patients were not randomised to lithium treatment. The patients who received lithium treatment were more often men (58.0% versus 42.0%, *P* = 0.03) and they received somewhat more ECTs during the index series (9.6 ± 4.1 versus 7.8 ± 2.9, *P* < 0.001).

#### 3.6.2. Antipsychotics during the Follow-Up Period

Nonpsychotic patients treated with antipsychotics had an increased risk of relapse/recurrence as compared to nonpsychotic patients treated without antipsychotics (HR =1.99, *P* < 0.001).

However, the patients were not randomised to treatment with antipsychotics. The patients who received antipsychotics were more often treated involuntarily (14.8% versus 3.5%, *P* < 0.001), and they were less often treated as outpatients (14.8% versus 32.4%, *P* = 0.002).

#### 3.6.3. Benzodiazepines during the Follow-Up Period

Patients treated with benzodiazepines during the follow-up period had an increased rate of relapse/recurrence as compared to patients without benzodiazepine treatment (HR = 1.43 *P* = 0.03). However, the patients were not randomised to treatment with benzodiazepines. Patients treated with benzodiazepines were more often treated for recurrent depression (88.9% versus 75%, *P* = 0.003), less often for psychotic depression (10.3% versus 23.1%, *P* = 0.021), and were more often diagnosed with anxiety disorders (40.4% versus 26.3%, *P* = 0.006).

#### 3.6.4. Continuation ECT

59 (12.1%) of the patients received continuation ECT during the follow-up period. The mean number of continuation ECT sessions was 12 ± 15. Continuation ECT was not associated with a significantly reduced rate of hospitalisation (HR = 0.83, *P* = 0.42). However, the patients were not randomised to continuation ECT. The patients who received continuation ECT more often had recurrent episodes (89.8% versus 76.6%, *P* = 0.02), they were more often treated as outpatients (49.2% versus 21.1%, *P* < 0.001), they more often had a comorbid anxiety disorder (40.7% versus 27.9%, *P* = 0.044), and they received somewhat more ECTs during the index series (9.5 ± 3.4 versus 7.9 ± 3.0, *P* < 0.001).

#### 3.6.5. Outpatient Care

Patients treated as outpatients during their index ECT had a much decreased risk of hospitalisation or suicide during followup as compared to patients receiving their index ECT as inpatients (HR = 0.43, *P* < 0.001).

### 3.7. Significant Factors Predicting Relapse

In a multivariate Cox regression analysis, the following factors were associated with increased or decreased risk for relapse/recurrence during the follow-up period.

**Decreased risk:**

outpatient treatment during the index ECT series.

**Increased risk:**

comorbid substance dependence, benzodiazepine treatment, and treatment with antipsychotics (Table 4).

## 4. Discussion

In this naturalistic setting, the cumulative proportion of patients with relapse/recurrence during one year after ECT was 35%. Thus, the risk of relapse/recurrence is high, in line with other studies where relapse/recurrence rates exceeding 30% have been reported [4, 5, 7–9, 21]. Suicide and hospitalisation are robust markers of relapse/recurrence. However, some patients may relapse into less severe symptomatology not requiring hospitalisation. Thus, we provide conservative estimates of the true rate of symptom reoccurrence. If relapse/recurrence had been based on symptomatic worsening (not hospitalization and suicide), as in the controlled trials by Sackeim and others [7–9], the relapse/recurrence rates would most likely have been higher. 

Patients with comorbid substance dependence had an increased risk of hospitalisation during followup. Some of those patients were hospitalised for other diagnoses than depression. It is possible that treatment of the comorbid diagnosis needs more attention, rather than intensified treatment for depression. The fact that comorbid conditions increase the risk of relapse/recurrence is in line with results from other studies [16, 17].

In major depression, a greater number of depressive episodes tend to increase the risk of subsequent relapses/recurrences [13]. However, in the present study, patients with major depressive disorder, recurrent type did not have significantly higher number of relapses/recurrences than the patients with major depressive disorder, single episode.

In some studies, severe symptoms have been associated with an increased risk of relapse/recurrence [18]. However, such a relationship is not supported from the results of the present study.

As concerns treatment strategies during the follow-up period, it is difficult to draw firm conclusions from naturalistic studies as the patients are not randomised and different factors related to selection might have influenced the results. However, it is remarkable that benzodiazepines and antipsychotics during the follow-up period seem to increase the risk for relapse/recurrence. As concerns antipsychotics, there are some results indicating a place for the second-generation antipsychotics in the acute treatment of mood disorders [11, 22]. In the present study, nonpsychotic patients treated with antipsychotics had an increased risk of relapse. The specific antipsychotic agents used were not registered, so the effect of different antipsychotics could not be compared. However, there is little information supporting the long-term use of antipsychotics after ECT in patients with major depressive disorder. Also, metabolic side effects with antipsychotics should be considered. Patients treated with benzodiazepines were at increased risk for relapse. Long-term treatment with benzodiazepines is not supported by existing guidelines. In addition, there is data suggesting that benzodiazepines may have a depression-inducing effect and the risk for iatrogenic substance dependence should be considered [23]. 

The present study gives some support to earlier findings indicating that lithium might prevent relapse/recurrence after ECT [7]. However, the patients were not randomised to lithium treatment. The proportion of patients receiving lithium differed markedly between the sites. Thus, it seems as if different treatment traditions in the hospitals account for most of the variation. Possible confounders to the preventive effect of lithium are a lower proportion of patients with benzodiazepine treatment and a higher proportion of men. Although these factors may contribute to the interaction, it is unlikely that the factors account for most of the effect. Also, in the multivariate Cox regression model, there was a tendency towards an effect of lithium for prevention of relapse/recurrence. Thus, the results indicate that more randomised controlled trials are needed to demonstrate if lithium is an alternative to prevent relapses/recurrences after ECT.

## 5. Conclusions

Our results demonstrate that relapses/recurrences resulting in psychiatric hospitalisation or suicide are common after ECT and should be a major concern even in remitted patients and patients treated for their first episode of depression. A one-year suicide risk of 2% is reported. The high risk of relapse/recurrence motivates intensive followup and treatment. Patients with substance dependence had increased risks of relapse after ECT. Treatments with antipsychotics, benzodiazepines, and continuation ECT were not associated with significant reductions in the risk for relapse/recurrence. Although the patients were not randomised to these treatments, the lack of effect indicates that better treatment strategies are needed. Lithium treatment seemed to be associated with a reduced risk of relapse. Thus, lithium needs to be further evaluated in post-ECT relapse prophylaxis of recurrent major depression.

##  Conflict of Interests

All authors declare no conflict of interests.

## Figures and Tables

**Table 1:** Patient characteristics.

		All patients *N* = 486
Sex	Men	43.5%
	Women	56.5%
Age, years	Mean ± SD	55 ± 18
Diagnoses	Major depression, single episode	22.3%
	Major depression, recurrent	77.7%
Severity	Mild/moderate	35.7%
	Severe, nonpsychotic	43.9%
	Severe, psychotic	20.4%
Type of care	Involuntary care	13.2%
	Voluntary care	86.8%
	Outpatient care	24.6%
	Inpatient care	75.4%
Comorbid axis 1 diagnosis	With comorbid anxiety diagnosis	29.5%
	With comorbid substance dependence	10.4%
Comorbid axis II diagnosis	With personality disorder	7.5%
CGI	Very much improved	29.8%
	Much improved	49.6%
	Minimally improved	13.8%
	Not improved	6.8%

**Table 2:** Univariate Cox regression analyses of patient risk factors for psychiatric hospitalisation or suicide during followup.

		Hazard ratio (HR)	95% Confidence interval (CI)	*P*
Sex	Women (*n* = 275) versus men (*n* = 211)	1.15	0.86–1.53	0.35

Age (years)		1.00	0.99–1.00	0.28

Diagnoses *N* = 486	Major depression, recurrent (*n* = 378) versus major depression, single episode (*n* = 108)	1.26	0.88–1.79	0.21

Severity	Mild/moderate (*n* = 168) versus severe, psychotic (*n* = 98)	1.01	0.67–1.52	0.97
	Severe, nonpsychotic (*n* = 210) versus severe, psychotic (*n* = 98)	1.12	0.76–1.66	0.57

Comorbid axis 1 diagnosis *N* = 486	With comorbid anxiety disorder (*n* = 140) versus without comorbid anxiety diagnosis (*n* = 346)	1.12	0.83–1.52	0.47
	With substance dependence (*n* = 50) versus without substance dependence (*n* = 436)	1.90	1.28–2.82	**0.001**

Comorbid personality disorder	With personality disorder (*n* = 37) versus without known personality disorder (*n* = 449)	1.41	0.88–2.26	0.16

CGI	Much improved (*n* = 229) versus very much improved (*n* = 136)	1.23	0.87–1.75	0.25
	Minimally improved (*n* = 63) versus very much improved (*n* = 136)	0.99	0.59–1.65	0.96
	Not improved (*n* = 31) versus very much improved (*n* = 136)	1.54	0.86–2.75	0.15

**Table 3:** Univariate Cox regression analyses of the influence of treatment factors on hospitalisation or suicide during followup.

		Hazard ratio (HR)	95% Confidence interval (CI)	*P*
Type of care	Outpatient (*n* = 119) versus inpatient (*n* = 367)	0.43	0.29–0.64	**<0.001**
	Involuntary (*n* = 63) versus voluntary (*n* = 423)	1.01	0.66–1.55	0.95

Patients with psychotic features	Antipsychotics (*n* = 54) versus no Antipsychotics (*n* = 42)	0.76	0.39–1.49	0.43

Patients without psychotic features	Antipsychotics (*n* = 81) versus no Antipsychotics (*n* = 293)	1.99	1.41–2.82	**<0.001**

Lithium treatment during followup	Lithium (*n* = 50) versus no Lithium (*n* = 429)	0.51	0.29–0.92	**0.03**

Benzodiazepines during followup	Benzodiazepines (*n* = 99) versus no Benzodiazepines (*n* = 380)	1.43	1.03–1.99	**0.03**

Continuation ECT during followup	Continuation ECT (*n* = 59) versus no Continuation ECT (*n* = 427)	0.83	0.53–1.30	0.42

**Table 4:** Multivariate Cox regression analysis of the influence of comorbid substance dependence, inpatient/outpatient status, lithium treatment, benzodiazepine treatment, and antipsychotics treatment on the risk for psychiatric hospitalisation or suicide during followup (*n* = 479).

At last step	Hazard ratio (HR)	95% Confidence interval (CI)	*P*
Outpatient	0.42	0.28–0.64	**0.00**
Substance dependence	1.88	1.26–2.80	**0.00**
Benzodiazepines	1.50	1.08–2.10	**0.02**
Antipsychotics	1.39	1.02–1.88	**0.04**
Lithium	0.58	0.32–1.02	0.06

	Model chi-square = 40.0, *df* = 5, *P* < 0.001		
